# Analysis of the relationship between liver regeneration rate and blood levels

**DOI:** 10.12669/pjms.311.5864

**Published:** 2015

**Authors:** Myeong-Seong Kim, Hae-Kag Lee, Seon-Yeong Kim, Jae-Hwan Cho

**Affiliations:** 1 Myeong-Seong Kim, PhD, Department of Radiology, National Cancer Center, Graduate School of Public Health and Institute of Health and Environment, Seoul National University, Republic of Korea.; 2 Hae-Kag Lee, PhD, Department of Computer Science and Engineering, Soonchunhyang University, Republic of Korea.; 3 Seon-Yeong Kim, PhD, Center for Proton Therapy, National Cancer Center, Department of International Radiological Science, Hallym University of Graduate Studies, Republic of Korea.; 4 Jae-Hwan Cho, PhD, Department of International Radiological Science, Hallym University of Graduate Studies, Republic of Korea.

**Keywords:** CT 3D Volumetry, Liver regeneration volume, Regeneration rate, Blood levels

## Abstract

**Objectives:**

The aim of this study was to investigate the difference of liver function changes according to the liver regeneration rate after liver transplantation through blood tests.

**Methods:**

Fifty donors, who underwent computed tomography (CT) 3D volumetry, were analyzed before and after liver transplantation. CT 3D volumetry was used as a study method to measure the mean liver regeneration volume and regeneration rate. Then, blood levels were measured including alanine transaminase (ALT), aminotransferase (AST), gamma-glutamyl transpeptidase (GGT) and total bilirubin.

**Results:**

The liver regeneration rate rapidly increased from 39.13±4.91% befoone1 month and 90.31±13.09% 16 months after surgery furthermore. Blood levels rapidly increased 7 days after surgery and then decreased 16 months after surgery compared to the state before surgery.

**Conclusion:**

This study results could be used as a basis for the prognosis of future liver transplantations.

## INTRODUCTION

Liver transplantation is the most effective treatment in patients with advanced end stage liver cirrhosis, metabolic liver disease, hepatocellular carcinoma, etc.^1^^-^^3^ A current liver transplantation mainly provides approximately from 60 to 65% of donor’s right lobe liver to recipient and its function recovers through volume increasing by left lobe liver regeneration. This kind of liver regeneration is the recovery process of liver parenchyma volume through the remaining liver cell proliferation as an essential component after damage. It is known that a normal liver shows regeneration and recovery after approximately 70-80% resection and the normal liver parenchyma of animals using rats shows an active regeneration after approximately 90% resection and liver function maintenance.^4^ Also the liver regeneration rapidly increased from within 72 hours to 2 weeks after resection. Later, it increased gradually and reached its maximum between 6 months and one year. Then it was recovered to more than 75% of the original volume.^5^^,^^6^

A right lobe donor resection provides enough functional volume to the recipient but it covers approximately two-thirds of the donor’s liver. In general, if the donor liver’s size is larger, the recipient’s effective treatment is better. However, the risk to get a liver failure is higher to the donor.^7^ Therefore, the number of studies for the prognosis of liver regeneration rate after right lobe liver transplantation is increasing for the safety of donors. According to Hagaet et al. the liver regeneration rate is recovered to 89.8% after 6 months of surgery and a longer the period was reported as safe.^8^ However, there was not enough data to prove that a higher liver regeneration rate means higher functional improvement. In general, liver function test is performed through blood tests. Clinically, alanine aminotransferase (ALT), aspartate aminotransferae (AST), alkaline phosphatase (ALP), total bilirubin (TB) and gamma-glutamyl transpeptidase (GGT) are commonly used among blood tests. In general, a blood test is a useful test as an index of hepatocellular damage and cholestasis as well as hepatic synthesis.^8^^-^^10^ There are no studies comparing the outcome of the different quantitative liver function tests within a standardized, clinically relevant model, although all the tests are clinically used.

Therefore, this study evaluates through blood tests the difference of liver function changes according to the liver regeneration rate after liver transplantation.

## METHODS

Fifty right lobe liver donors (31 male and 19 female) were analyzed who visited the hospital and underwent a liver transplantation from January 2011 to December 2012.The suitability of liver transplantation was determined by a CT Hepatobiliary-CTA test KH. Anda 3DVolumetry before surgery and donor data were analyzed by abdomen CT test 7 days, one month and 16 months after liver transplantation. All participants signed written informed consent form approved by the Institutional Review Board at the National Cancer Center. The donors were between 16 and 50 years old and the mean age was 29.1 years. The images were acquired using existing data. The CT test equipment was Brilliance 64 (Philips Medical System, Netherland). The liver regeneration was measured by 3D Volumetry using Mx View V3.52 (Philips Medical System Netherland) to assess how the volume and its rate of donor was increased before and after the liver transplantation. The portal phase protocol of CT Hepatobiliary-CTA and Abdomen CT of the hospital was used as test protocol. Portal phase parameter is as follows (Table-I). The data measurement of the donor before liver transplantation was analyzed after division by 3 mm interval of total liver at portal phase with the sum of value after 3D Volumetry at workstation among CT Hepatobiliary-CTA. Data after liver transplantation were analyzed with the sum of value after 3D Volumetry at workstation after division by 3 mm interval at 7 days, 1 month and 16months after surgery. In particular, an investigator manually measured the data to maintain the objectivity of 3D Volumetry.

**Liver regeneration rate (%) = (Post op liver volume / Pre op liver volume) × 100(1)**

Next, subjects were tested of ALT, AST, GGT and total bilirubin after fasting for at least 12 hours or more. Four kinds of liver function blood test are frequently used as an indicator related to liver function. In addition, the volume density of liver is 1.12 which is similar to the water density 1. Therefore, the increased volume and regeneration rate were measured with the density of 1 at data analysis in 3D Volumetry statistically data analysis. The difference of liver regeneration volume, regeneration rate and blood test was tested using the ANOVA test. After the ANOVA test, the Dunnett test was performed to more accurate differentiate for post-hoc analysis. In addition, the relationship between liver regeneration rate and blood levels was tested using linear regression analysis. Statistical analysis used SPSS ver. 18.0 for software with a significance level less than 0.05.

## RESULTS

As the result of liver regeneration volume measurement using 3D Volumetry, the mean volume was 524.30±129.37 cc before liver transplantation and increased to 717.38±139.16 cc at 7 days, to 917.63±152.96 cc at 1 month and to 1197.59±256.87 cc at 16 months after surgery (Table-II) (p<0.05).The liver regeneration rate was 39.13±4.91% before surgery and rapidly increased to 54.07±5.65% 7 days after surgery, then increased gradually to 69.67±8.92% one month and 90.31±13.09% 16 months after surgery. The liver regeneration rate could be represented as linear = 0.049 x date - 0.575 and the explanatory power was 82.2% (p<0.05). And finally, 100% regeneration rate is expected approximately 30 months after surgery. As result of mean blood levels analysis per date, AST was 22.26±15.03 u/L before surgery and rapidly increased to 54.98±16.21 u/L 7 days after surgery. Then it decreased to 23.58±14.49 u/Lat 16 months after surgery (p<0.05). ALT was 16.54±7.79 u/L before surgery, rapidly increased to 75.06±26.28 u/L 7 days after surgery and decreased to 19.80±16.99 u/L 16 months after surgery (p<0.05).GGT was 23.24±16.20 u/L before surgery and rapidly increased to 54.49±29.85 u/L 7 days and 68.30±118.37 u/L one month after surgery. Then it decreased to 33.40±64.27 u/L 16 months after surgery (p<0.05). Bilirubin was 0.30±0.07 mg/dl before surgery and rapidly increased to 2.21±5.19 mg/dl after 7 days. Then it decreased to 0.96±0.40 mg/dl after 16 months (Table-III) (p<0.05). As a result, blood levels rapidly increased 7 days after surgery compared to the results before surgery and then decreased after 16 months recovery to the levels before surgery (Fig.2). The levels of AST, ALT and bilirubin did not linearly decrease or increase in the linear analysis of liver regeneration volume and blood test levels. However, the GGT level could be represented as linear of Liver regeneration volume = 0.719 × GGT value + 880.40.The explanatory power was 35.5%. It means liver regeneration volume increases as GGT increases (Fig.3). The bilirubin value didn’t linearly decrease in the linear analysis of liver regeneration rate and blood test values. However, GGT levels could be represented as linear of Liver regeneration rate = 0.046 × GGT value + 67.78 and the explanatory power was 40.0% (Table-IV). As a result, the liver regeneration rate increases as GGT value increases (Fig.3).

## DISCUSSION

Liver transplantation is the optimal treatment for cirrhosis and hepatocellular carcinoma. However, the availability of cadaveric organs is absolutely lacking compared to domestically waiting patients and so living donor liver transplantations replace cadaveric organ transplantations.^12^^,^^13^ In live donors, safety is always a top priority for liver transplantation.^14^ However, a donor provides its right lobe of liver to meet metabolic needs of the recipient to patients with a general liver failure. After the right lobe resection the donor should maintain the liver function with the left lobe only which could lead to safety problems. In addition, it could impact the prognosis of donor or recipient in case of a liver regeneration failure.^15^ In general, blood tests are performed for liver function test. ALT, AST, ALP, TB and GGT are clinically used among liver blood tests.^9^

In this study, the liver function change was evaluated according to the liver regeneration rate after liver transplantation. The study showed that ALT, AST, TB and GGT levels rapidly increased at 7 days after surgery compared to the state before surgery and then decreased at 16 months after surgery recovering compared to the state before surgery. ALT and AST levels are related to the liver parenchymal cells. Liver parenchymal cells are destroyed directly by microorganism and could be modified by immunological causes.^16^ It is reported that enzyme activities such as ALT and AST are increased if liver parenchymal cells are modified. Enzymes involved in amino acid metabolism exist in hepatocytes and its blood concentration could be increased. When hepatocytes are damaged then enzymes are released to blood.^17^ In this study, the rapid increase at 7 days after surgery is considered to be triggered by liver parenchymal cell’s modification and an increased enzyme activity (p=0.003). In addition, liver cells are recovered as time goes on so that ALT and AST are considered to be decreased. Bilirubin is produced as an unconjugated form by hemoglobin after the end of life of red blood cells from the reticuloendothelial system such as in the spleen. The unconjugated form of bilirubin is not hydrophilic and is excreted through the biliary tract after switching to a hydrophilic conjugated form by bilirubin-UDL-glucuronosyltransferase (BUGT) in liver cells. When liver is damaged by infection, bilirubin is reported to be increased.^18^

**Table-I:** Scan parameter of portal phase

**Parameter**	**Portal Phase**	
	**CT Hepato Biliary/CTA**	**Abdomen CT**
Kv	120	
Ma Range	250-446	
Thickness	3	
Increment	3	
Collimation	40mm	
Pitch	0.891	
Rotation Time	0.5sec	
Injection Volume/ Flow Speed	120cc / 4.0ml	120cc/2.0ml

**Table-II:** Mean liver regeneration measurement by date

	**Liver regeneration (cc)**	**Liver regeneration rate (%)**
Total Volume	1337.12±279.00	-
Preoperative	524.30±129.37	39.13±4.91
7 days after operative	717.38±139.16	54.07±5.65
1 month after operative	917.63±152.96	69.67±8.92
16 months after operative	1197.59±256.87	90.31±13.09

**Table-III:** Mean blood test results by date

	**AST (u/L)**	**ALT (u/L)**	**GGT (u/L)**	**Bilirubin (mg/dl)**
Preoperative	22.26±15.03	16.54±7.79	23.24±16.20	0.30±0.07
7 days after operative	54.98±16.21	75.06±26.28	54.49±29.85	2.21±5.19
1 month after operative	27.06±8.69	27.62±13.36	68.30±118.37	0.80±0.35
16 months after operative	23.58±14.49	19.80±16.99	33.40±64.27	0.96±0.40
P	0.000	0.000	0.006	0.003

**ALT:** alanine transaminase, **AST:** aminotransferase, **GGT: **gamma-glutamyl transpeptidase.

**Table-IV:** The linear analysis of liver regeneration volume and rate, blood levels

**Model**		**Non-** **standardized ** **coefficients** ** (B)**	**P**
Liver Regeneration	Constant	880.403	0.000
	AST	-1.432	0.463
	ALT	-0.868	0.512
	GGT	0.719	0.029
	Bilirubin	2.348	0.774
Liver Regeneration Rate	Constant	67.789	0.000
	AST	-.127	.337
	ALT	-.078	.385
	GGT	.046	.042
	Bilirubin	.240	.666

**ALT:** alanine transaminase, **AST:** aminotransferase, **GGT: **gamma-glutamyl transpeptidase .

**Fig.1:**
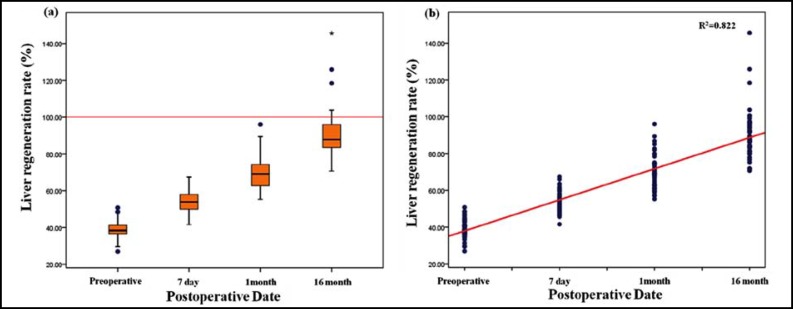
Liver regeneration rate after surgery by date (a) and the linear analysis graph of regeneration rate (b).

**Fig.2:**
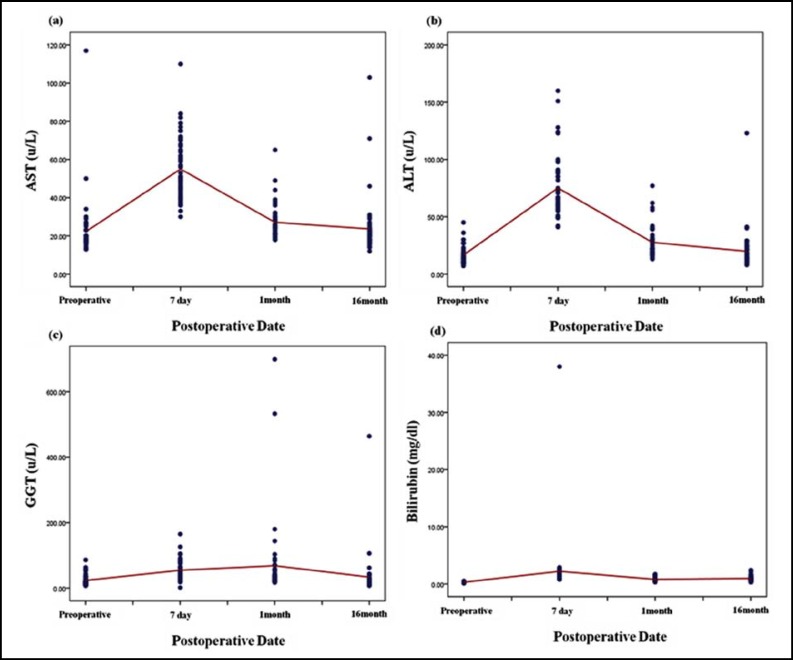
Analysis of aspartate aminotransferase (a), alanine aminotransferase (b), gamma-glutamyl transpeptidase (c), total bilirubin (d) after surgery by date.

**Fig.3:**
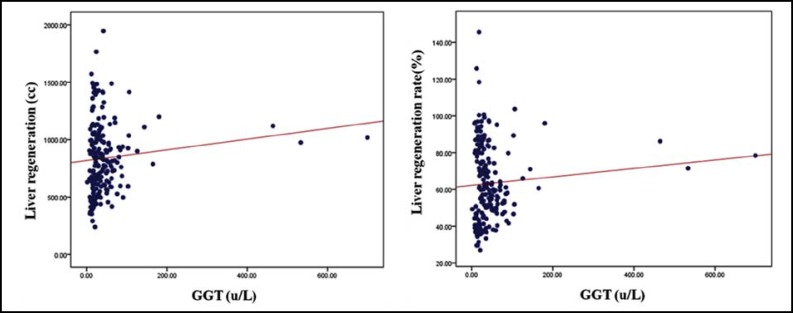
The linear graph of liver generative volume and gamma-glutamyl transpeptidase value

 As a result of this study, bilirubin is considered to be increased due to liver damage after 7 days. Additionally, GGT greatly increased in mainly bile congestive liver disease and slightly increased less than 3 times than normal.^18^ Above results are considered to be increased by liver damage. According to previous studies and our result, liver function values such as AST, ALT and PT showed their maximum in all groups at 2 days after surgery and then decreased gradually suggesting that liver recovery occurs just after surgery.^19^^-^^21^ Conclusively, this study suggests that ALT, AST, TB and GGT values decrease as time goes on which means the liver recovers with time. This study analyzed blood levels according to liver regeneration in patients with liver transplantation. The study results are considered to be used as a basis for the prognosis of future liver transplantations.
